# Supplementary figures and images for: The anti-platelet drug cilostazol enhances heart rate and interrenal steroidogenesis and exerts a scant effect on innate immune responses in zebrafish
Source: PLoS One. 2023 Oct 30;18(10):e0292858. doi: 10.1371/journal.pone.0292858 (PMC10615288; doi:10.1371/journal.pone.0292858)

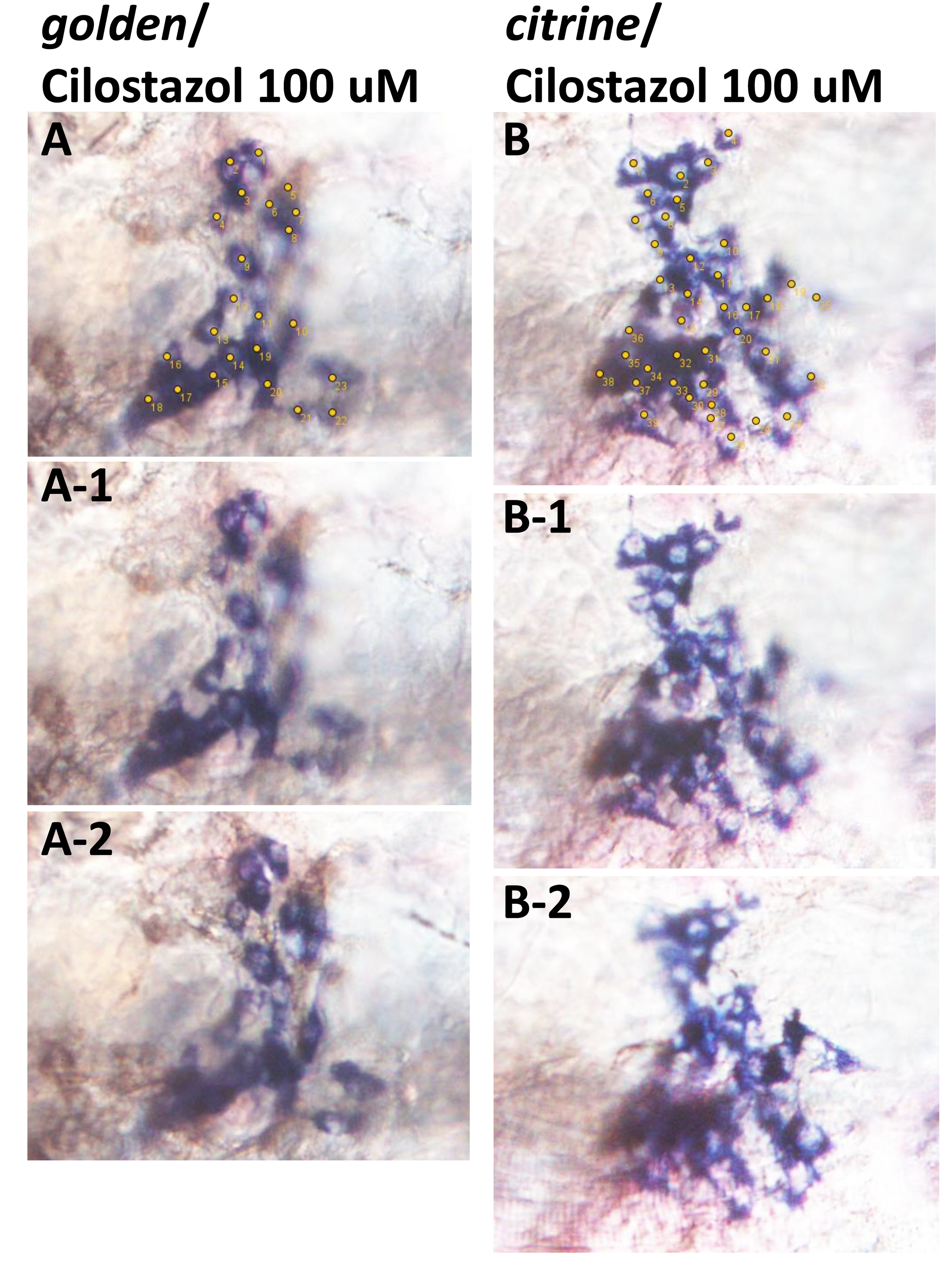

Supplement: S1 Fig — The representive images of golden (A, A1, A2) or citrine (B, B1, B2) embryos treated with 100μM cilostazol respectively as in Fig 2 are shown as examples. Panels (A, B) demonstrate the cell counting by ImageJ. (A1, A2, B1, B2) In the cases where stained interrenal cells on the ventral surface need to be identified by adjusting the focus, multiple images were taken in order to provide sufficient resolution for the whole interrenal tissue clusters. (TIF) [file pone.0292858.s001.tif]

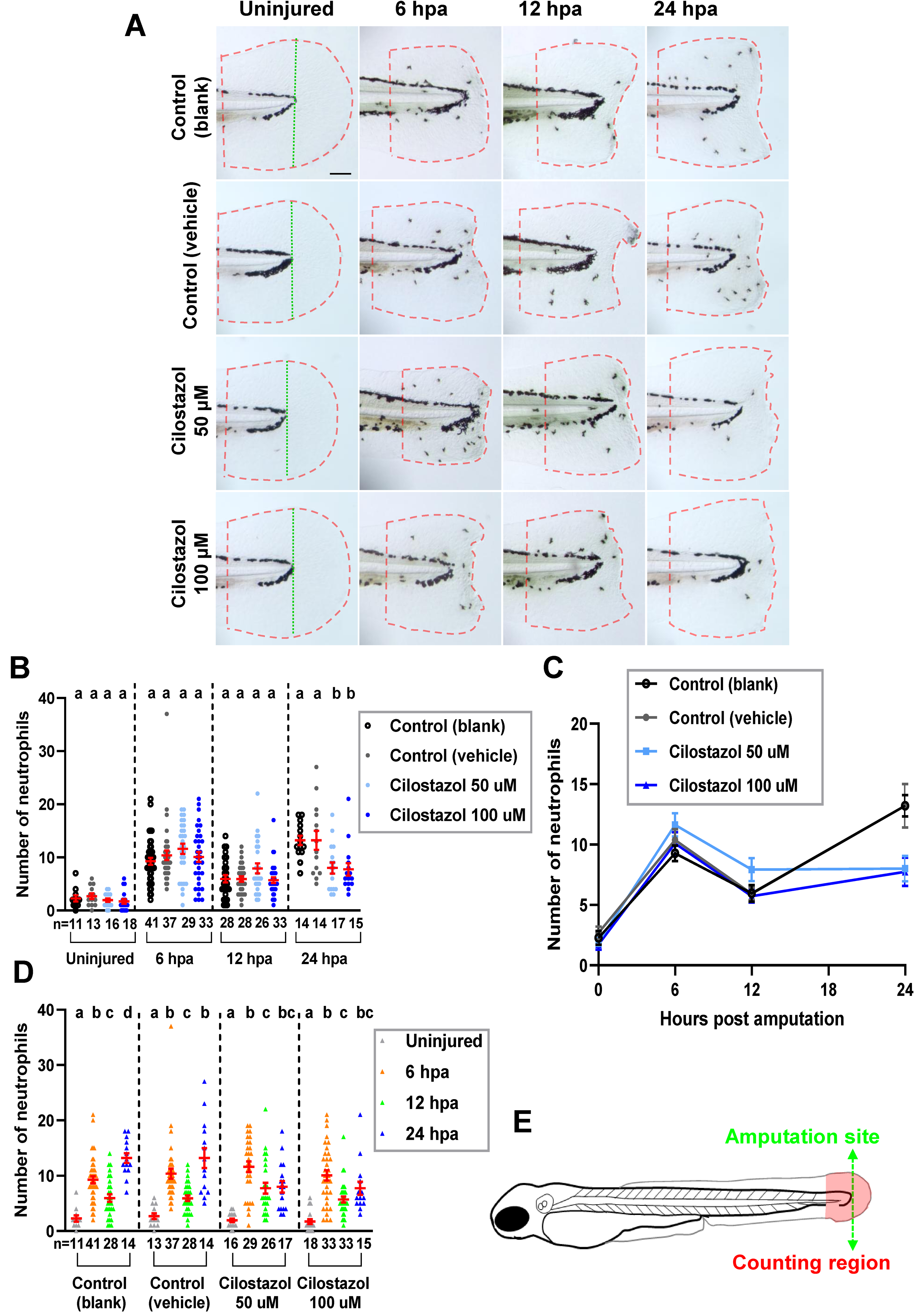

Supplement: S2 Fig — (A) The embryonic zebrafish were subject to treatments of cilostazol at 50 and 100 μM respectively, or blank and vehicle controls, from 24 hpf onwards to the time of harvest. The amputation of caudal fin fold was performed on the zebrafish embryos at 3 dpf, which were subsequently fixed at 6, 12 and 24 hpa respectively for whole-mount Mpx enzymatic staining. The uninjured fins at 3 dpf were also stained to show the background level of neutrophils prior to their migration to the wounded region. The green dotted line indicates the site of resection, and the area in-between levels of the anterior edge of pigment gap and the posterior edge of regenerating fin, highlighted by red broken lines, was selected for neutrophil quantification. The amputation site and neutrophil counting region are also depicted in the schematic diagram in (E). (B) A comparison of neutrophil accumulation in the selected area of counting as shown in (A), among different treatment groups at the uninjured fins; or at regenerating fins at 6, 12 and 24 hpa respectively. At each time point, differences in the number of neutrophils were compared among various treatment groups. Columns of data with different letters above them are significantly different (Kruskal-Wallis analysis followed by Dunn’s test, P < 0.05). (C) A line chart showing the temporally dynamic changes of neutrophil accumulation in the wounded fin area, in different treatment groups as shown in (B). (D) A comparison of neutrophil accumulation in the wounded fin area among different time points after fin amputation, in each treatment group as shown in (A). Kruskal-Wallis followed by Dunn’s test was performed for the analysis of blank and vehicle control groups. Welch’s ANOVA followed by Games-Howell test was performed for the analysis of 50 and 100 μM cilostazol treated groups. Columns of data with different letters above them are significantly different (P < 0.05). (TIF) [file pone.0292858.s002.tif]

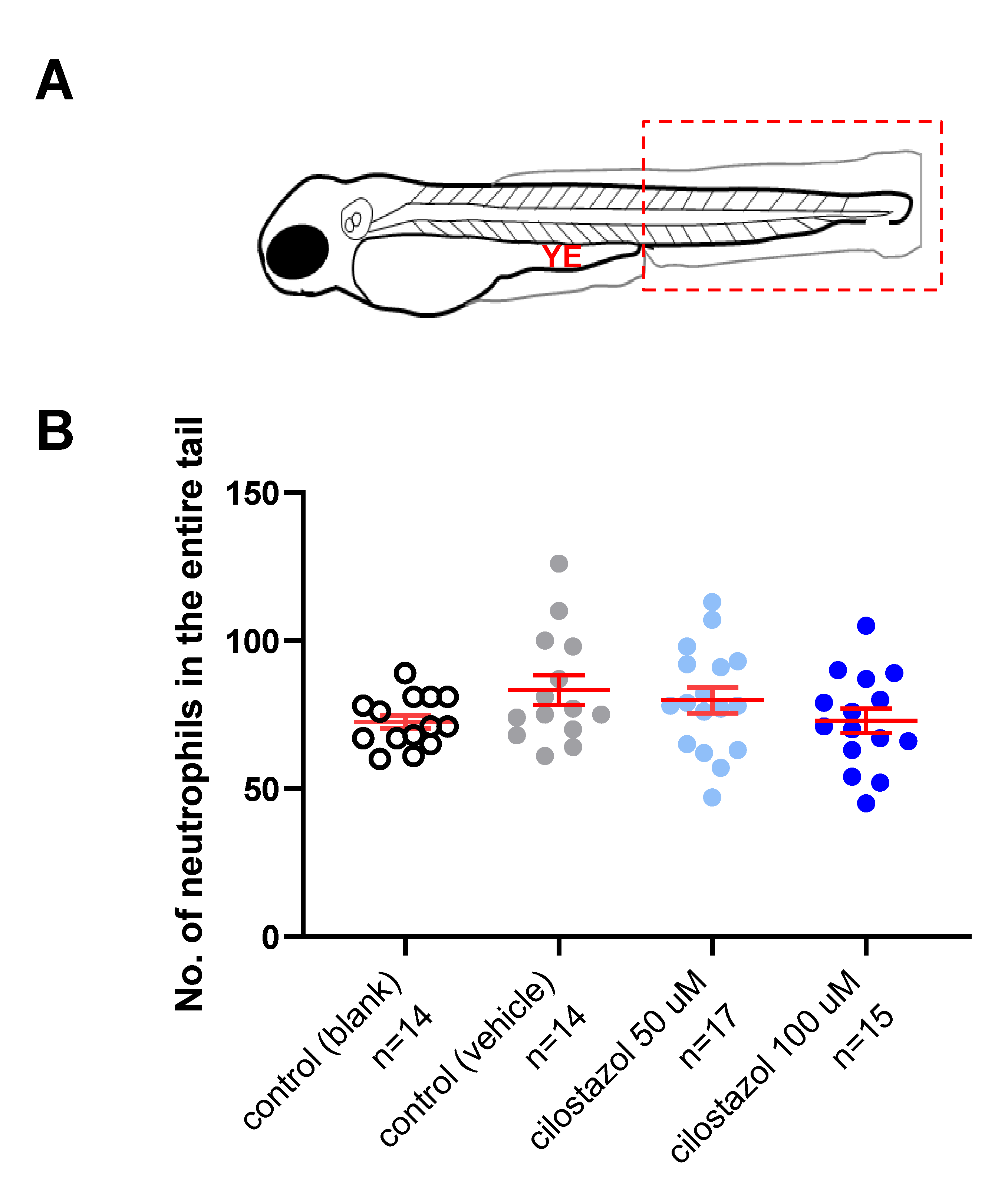

Supplement: S3 Fig — (A) The area used to determine the total amounts of neutrophils present in the whole tail region (posterior to the yolk extension; YE) is indicated by a red dashed box. (B) A comparison of neutrophil accumulation in the counting area as shown in (A) among different treatment groups at 24 hpa, where no statistical difference is observed (ANOVA and Tukey’s multiple comparisons test). (TIF) [file pone.0292858.s003.tif]
