# Supplementary material for: The anti-platelet drug cilostazol enhances heart rate and interrenal steroidogenesis and exerts a scant effect on innate immune responses in zebrafish
Source: PLoS One. 2023 Oct 30;18(10):e0292858. doi: 10.1371/journal.pone.0292858 (PMC10615288; doi:10.1371/journal.pone.0292858)
Supplement: Attachment — Submitted filename: plos1 review.pdf [file pone.0292858.s007.pdf]

Summary:

The authors of this manuscript seek to investigate the effects of the drug Cilostazol on steroidogenesis and immune cell activity using larval zebrafish as a model. Cilostazol is a PDE3 inhibitor that generally causes an increase in cellular cAMP levels in affected tissues by preventing metabolism to AMP, which may alter physiology. The authors treat larval zebrafish with this drug and validate is efficacy by verifying it can increase zebrafish heart rate. They then provide evidence to suggest that treatment of the drug can alter steroidogenesis by increasing the number of active interrenal cells in the fish and increasing whole-body cortisol levels. The authors then investigate cilostazol-mediated changes in immune cell activity by utilizing a tail fin clip injury model. While tail fin regrowth and macrophage recruitment was not affected by drug treatment, the authors claim that neutrophil migration and retention in the injury site is altered. The authors interpret this data to suggest that cilostazol can adversely modulate sterile inflammatory processes during development and caution against treating young developing patients with the drug.

Abstract

- Major Issues
- Minor Issues

Introduction

- Major Issues
- Minor Issues
    - Line 68: "…the stress response hormone catecholamine". Catecholamines are a class of molecule with an aromatic catechol and amine side-chain most notably including dopamine, epinephrine, and norepinephrine. Chromaffin cell granules contain mostly epinephrine and norepinephrine along with some dopamine (https://doi.org/10.1016/B978-0-12-416687-5.00030-0). Authors should revise their language to reflect the stress response hormones are epinephrine and norepinephrine, not "catecholamine".
    - Line 104 "It is generally accepted…" Please provide a citation.

Methods

- Major Issues
    - Lines 135-6: Please verify that larval heartrate was recorded on high-speed video and analyzed offline in a slower playback. A human cannot accurately measure the reported heart rates between 90 and 230 bpm (1.5-4Hz). Authors should also describe in detail how a single heartbeat was quantified.
    - Line 121 Animals: Please provide references to the *golden* and *citrine* strains utilized.
- Minor Issues
    - Line 129: Authors should describe what percentage (maximal) of DMSO zebrafish are exposed to. High DMSO concentrations can affect heartrate.

Results

- Major Issues
  - Lines 213-15: "…enhanced in a dose-dependent manner from 30 to 100 µM, while the heart rates of 10 and 30 µM treatment groups did not show significant difference." The authors claim 30 µM cilostazol increases heart rate, but then claim it and 10µM had no significant difference (from untreated?). The authors should clarify if only 50 and 100 µM caused a significant increase.
  - Lines 213-15: It is unclear what control represents. Authors should compare treated groups to equivalent vehicle control groups.
  - Lines 248 – 250: Authors note that a low DMSO concentration increased measured cortisol levels. This accounts for approximately 30% of the claimed cortisol increase caused by 50 and 100 µM, a large amount. Additionally, the authors show that the DMSO control is significantly larger than the 30µM treatment group. The authors must address this difference. It is possible that inappropriate statistics are being used, or there is major batch variation causing false positive results. (See figure 3 comments).
  - Lines 281-2: See figure 4 comments. Duplicated data.
  - Lines 283-290: The authors have transformed their neutrophil stain data into a timeseries format. They mention a lower number of neutrophils present in all samples by 12hpa and another upregulation by 24 hpa. The authors use this data to claim cilostazol plays a role in neutrophil retention but not recruitment. However, previous studies using live tracking of neutrophil migration have not seen this resurgence phenomenon (PMID: 16926288) including a real-time live tracking study (PMID: 25763660). The authors should address this discrepancy. The authors should also consider performing a real-time live neutrophil tracking experiment in order to support their claim of cilostazol-mediated neutrophil retention.
  - Lines 292-304: The authors should make specific mention to the actual data-containing figure 5.
  - Lines 314-25: Why does DMSO vehicle significantly increase macrophage recruitment over blank control? As with the neutrophil staining, the authors should consider a live real-time analysis of macrophage migration to discuss trends.
- Minor Issues
  - Line 218: "…developing zebrafish may be similar to those on the mammalian adults." Strange phrasing. Rephrase to just say "similar in mammals".
  - Line 224: "…cilostazol, which enhances cAMP production…". Cilostazol increases intracellular cAMP concentration by blocking PDE3-mediated degradation. It is not known to directly increase cAMP production. Any literature showing cilostazol-mediated cAMP production should be cited.
  - Lines 240-241: The authors describe the cortisol extraction and quantification procedure in the methods, but they should also briefly mention the experiment again here before discussing results.

- Lines 251-53: The authors are trying to compare their cortisol findings with their steroidgenic cell counts in an unclear way. They may wish to create a new last sentence stating something along the line of “increased cortisol levels are consistent with increased number of steroidgenic cells”.

Discussion

- Major Issues
- Minor Issues
  - Lines 347-8: “Cortisol is the most important endogenous glucocorticoid in both humans and teleosts”. This is a completely subjective statement.
  - Line 351: Missing period.
  - Lines 398-400: The authors are slightly over stating their findings. Their study does not investigate the persistence of chronic inflammation into zebrafish adulthood, nor does it investigate changes in mental function of the fish. The authors should revise somewhat.

Figures

- Major Issues
  - Figure 2:
    - In panel A, the authors show stained tissue from two zebrafish pigmentless strains with varying cilostazol treatment. The authors have outlined the stained tissue with a red dashed line, but claim to have quantified the number of cells within the tissue in panels B and C. From the images provided, it is unclear how authors were able to resolve individual cells. The bottom center and rightmost images are particularly low resolution and blurry with large overlapping purple areas where individual cells do not appear to be resolvable. The authors must describe the quantification process in the methods. Additionally, the authors should provide sample images of individually identified cells outlined in a similar fashion to panel A, perhaps as a supplemental figure.
    - Panels B and C should have a label/title describing from which strain the data is coming from.
  - Figure 3:
    - In the figure legend, authors claim each sample size is a batch of 30 larvae with 3 samples per treatment group. They also mention either samples or larvae groups are “subject to duplicate measurements”. Is each duplicate graphed here as an independent datapoint creating N = 6 samples? This would artificially bias the data. If each histogram is only N = 3, is this enough data points to make statistical conclusions?
  - Figure 4:
    - **The authors provide a figure showing that cilostazol treatment causes fewer migrated neutrophils at 24 hpa. However, the 24 hpa provided images in Panel A of 50 and 100 µM treatment have been duplicated.

Despite the images being duplicated, the red traced outline is slightly different between the two. The authors should explain and correct the error and ensure that duplicated data was not shared between the 50 and 100 µM dot plots.

        - 452: "50 and 100 µM cilostazol control groups". These are not control groups.

- Minor Issues
    - Figure 5 is not mentioned explicitly in the text.
