# Supplementary material for: The anti-platelet drug cilostazol enhances heart rate and interrenal steroidogenesis and exerts a scant effect on innate immune responses in zebrafish
Source: PLoS One. 2023 Oct 30;18(10):e0292858. doi: 10.1371/journal.pone.0292858 (PMC10615288; doi:10.1371/journal.pone.0292858)
Supplement: Attachment — Submitted filename: Liu 2023.6.12 PLoS One Rebuttal letter.pdf [file pone.0292858.s008.pdf]

On behalf of the authors, I would like to thank the editor and the two reviewers for taking precious time to offer critical comments and helpful advice on this article. According to the suggestions, we have performed two new experiments to replace the previous Figure 1 and 4. Three supplemental figures that include additional analyses have also been added to this revised article. Our responses to the reviewer comments are detailed below.

Amendments Made in Response to Reviewer 1 Comments

Major points:

Does cilostazol effect total neutrophil number or neutrophil biogenesis? additionally, this result could be strengthened by using a full GFP reporter (neutrophil, lyzC) besides a granulo protein mpx, which could also be done by time lapse. This could also shed light on the decrease in the initial phase of neutrophil recruitment decrease seen at 12hpi. Furthermore, since the claim of less neutrophil recruitment is of a retention phenotype, I would suggest a LTB4 bath model to assess neutrophil swarming to test this hypothesis.

Response

We thank Dr. Hsu for raising an important question as to whether cilostazol affects total neutrophil number or neutrophil biogenesis. Based on the experiment of Mpx-activity staining assays, we have further analyzed the total amounts of neutrophils in the entire tail area (Fig. S3), in order to verify whether cilostazol specifically suppresses the retention of neutrophils in the wounded region. Our results indicate that cilostazol does not influence the total neutrophil number in the entire tail, which has been described in lines 342-6.

We do agree with Dr. Hsu's comment that our results could be further strengthened by using the transgenic lyzC reporter besides the Mpx enzymatic activity assay. We apologize for not being able to carry out the lyzC reporter assay due to unavailability of the line in our local zebrafish community. Nevertheless, we have added one more experiment using the *Tg(mpx:GFP)* line to estimate the influence of cilostazol on the neutrophil migration (Fig. 4), where a decrease in the initial phase of neutrophil recruitment was also seen at 12 hpi/hpa. The previous Fig. 4 results based on the whole mount Mpx activity assay have been moved to Fig. S2 for comparisons. The results of and discrepancy between these two experiments are described in the Results (lines 310-359). We found that the trend of neutrophil dynamics and the effect of cilostazol treatments appeared divergent between *mpx:EGFP* live imaging and Mpx enzymatic activity staining assays. It is speculated that different genetic backgrounds or various physiological conditions due to experimental processing might cause the difference.

We also appreciate the suggestion about using LTB4 model to corroborate our neutrophil migration results. Indeed leukotriene B4 (LTB4 synthesis) is shown to display a 3 fold increase at 4 days post injury (dpi) in the zebrafish fin amputation model, accompanied by an increase of *alox5ap* mRNA expression [PMID: 27219276]. While the synthetic glucocorticoid beclomethasone

suppresses Mpx-positive neutrophils at both recruitment and retention stages, it inhibits the LTB4 synthesis at 4 dpi without affecting mRNA expressions of those genes involved in LTB4 biosynthesis. Therefore, the regulation of LTB synthesis by glucocorticoids may possibly not occur at the transcriptional levels. Apart from regulating gene expressions at the transcriptional levels, glucocorticoids are also known to exert post-transcriptional and nongenomic effects (PMID: 16113443). In our results, the effect of cilostazol on the neutrophil migration appears to be much more mild and subtle than that of the beclomethasone treatment. Moreover, othe results based on the *mpx:EGFP* reporter showed an effect of cilostazol at the recruitment rather than retention of neutrophils post injury (Fig. 4). In addition, lipoxin A4 (LXA4) biosynthetic pathway may also be a possible target for checking whether cilostazol affects chemoattractants for leukocyte migration. Hence, we consider that it may require another comprehensive study to reveal any possible effect of cilostazol on chemoattractant biosynthesis and those genes involved.

Discuss the neutrophil specific cilostazol effect, as a cortisol induced effect usually is accompanied by less initial recruitment

<u>Response</u>

We appreciate Dr. Hsu for reminding us that a cortisol induced effect usually is accompanied by less initial recruitment. Indeed, our new results carried out by *mpx:EGFP* reporter revealed a mild suppressing effect of cilostazol at 50 uM on neutrophil recruitment (at 6 hpa) rather than neutrophil retention (at 24 hpa). While the trend of neutrophil dynamics and the effect of cilostazol treatments appeared different between *mpx:EGFP* live imaging and Mpx enzymatic activity staining assays, we have tried to address the discrepancy in the Results (lines 310-359).

Although the increased cortisol production due to cilostazol treatments might contribute to the mild suppressing effect of cilostazol on neutrophil migration, it is not to be ruled out that cAMP levels might also affect the neutrophil behavior in the zebrafish fin amputation model. cAMP is known to regulate the migration of neutrophils in a complex and dynamic way. cAMP promotes neutrophil migration by regulating cytoskeletal elements through PKA activation (PMID: 21145500). However, another study suggests that cAMP inhibits neutrophil migration by disrupting polarity and chemotaxis (PMID: 26905427). Specific for the fin amputation model in zebrafish, it awaits future research to investigate any possible combined impact of cortisol and cAMP on the neutrophil migration. This paragraph has also been add to the Discussion (lines 457-464).

qRT of pro/anti inflammatory genes to help elucidate the mechanism for this decreased neutrophil recruitment

<u>Response</u>

We fully agree with Dr. Hsu that gene expression analysis of pro/anti-inflammatory genes by qRT-PCR will help elucidate the mechanism for the decreased neutrophil recruitment in our work.

A reported microarray study has shown a significant regulation of 31 immune-related genes at 4 hpi in the embryonic fish subject to fin amputation at 3 dpf [PMID: 27219276]. The synthetic glucocorticoid beclomethasone suppresses the expression of 27 and enhances the other 4 immune-related genes regulated by amputation. The potency of beclomethasone in terms of glucocorticoid activity is 53% that of dexamethasone (PMID: 25808113), while dexamethasone is considered 25-fold more potent than cortisol (PMID: 36308022). In our study, the cilostazol treatments led to mild effects on both cortisol production and neutrophil mobilization (Fig. 3, 4, S2). Therefore, the panel of 31 immune-related genes as mentioned above may need to be analyzed systematically in order to reveal any possible differences. On the other hand, the cilostazol effects on the neutrophil behavior may be influenced by experimental conditions or genetic backgrounds (as discussed in lines 310-359 of the revised article). As such, a more elaborate design including different time points post injury; and even various genetic backgrounds, may be required for the quantitative analysis of how cilostazol affects the repertoire of amputation-induced gene expressions.

On the other hand, cAMP increase due to cilostazol treatments might also influence neutrophil migration by regulating cytoskeletal elements and cell polarity. As mentioned above, any possible combined impact of cortisol and cAMP on the neutrophil migration due to cilostazol treatments awaits future investigation in the fin amputation model. Considering the complexity of these issues, we suppose that this might take another study to accommodate all the experiments. Nevertheless, the mechanistic study based on quantitative analysis of amputation-induced gene expressions will for sure shed light on how cilostazol influences neutrophils in the sterile injury.

I would recommend to tone down the discussion in terms of clinical or developmental correlations, as there is not enough evidence to support the extrapolations.

Response

We thank Dr. Hsu for this advice. In lines 384-6 of our original version: " However, our study suggests potential adverse endocrine effects of cilostazol for use in developing young patients, which need to be carefully assessed." has been revised as " However, our study suggests potential adverse endocrine effects of cilostazol for use during developmental stages, which need to be carefully assessed." (Lines 470-2 of revised version).

In the lines 398-400 of our original version: "Therefore, the results of our study implicate that exposure to cilostazol during early development may lead to pro-inflammatory adults with changes in mental function, which should be considered in the treatment of young patients." has been revised as "...the results of our study implicate that exposure to cilostazol during early development may lead to pro-inflammatory adults with changes in mental function, which remains to be investigated in animal models." (Lines 484-6 of revised version).

Minor points:

clarify annotation for a,b,c,d,e,f ect. on the figures
there is no Fig 5. corresponding text (should be in the section regarding no effect on tissue regeneration)

Response

We have described in the figure legends that the different letters above histograms in the bar chart, or above columns of data in the column scatter plot, serve as indicators of significant differences in multiple comparisons.

In addition, the data-containing Fig. 5A and B have been specifically mentioned in the revised version (lines 372-5).

<u>Amendments Made in Response to Reviewer 2 Comments</u>

Introduction

- Minor Issues
  - o *Line 68: "…the stress response hormone catecholamine". Catecholamines are a class of molecule with an aromatic catechol and amine side-chain most notably including dopamine, epinephrine, and norepinephrine. Chromaffin cell granules contain mostly epinephrine and norepinephrine along with some dopamine (https://doi.org/10.1016/B978-0-12-416687-5.00030-0). Authors should revise their language to reflect the stress response hormones are epinephrine and norepinephrine, not "catecholamine".*
  - o *Line 104 "It is generally accepted…" Please provide a citation.*

<u>Response</u>

1. Line 69 (line 68 in the original version): The original reference that we cited [PMID: 12688393] has used the term "catecholamine" because the authors actually measured a combination of epinephrine and norepinephrine by using the high-performance liquid chromatography. Therefore, we will follow this reference and use the term "catecholamine" added with (epinephrine + norepinephrine) in the revised version for clarity.
2. Line 106 (104 in the original version): The sentence has been revised with a reference added.

Methods

- Major Issues
  - o *Lines 135-6: Please verify that larval heartrate was recorded on high-speed video and analyzed offline in a slower playback. A human cannot accurately measure the reported heart rates between 90 and 230 bpm (1.5-4Hz). Authors should also describe in detail how a single heartbeat was quantified.*
  - o *Line 121 Animals: Please provide references to the golden and citrine strains utilized.*

<u>Response</u>

1. Lines 145-57 (lines 135-6 in the original version): We thank this reviewer for pointing out the drawback of our previous method for heart rate measurement. A new set of experiment has been performed using the ImageJ TSA approach based on what is described in (https://doi.org/10.3390/inventions3020021; https://doi.org/10.3390/biom11101476), and therefore Fig. 1 together with the related methods and results have been revised (lines 145-157; 240-55).
2. Lines 126-7: The references of the *golden* and *citrine* strains have been provided.

- Minor Issues

o *Line 129: Authors should describe what percentage (maximal) of DMSO zebrafish are exposed to. High DMSO concentrations can affect heart rate.*

Response

Line 137 (line 129 in the original version): A sentence describing the concentration of DMSO (0.18%) contained in the vehicle control solution has been added to the Materials and Methods. DMSO concentrations up to 1% are generally considered safe in developmental toxicity studies [PMID: 35295145], and how the concentration of DMSO at 0.18% affected embryonic heart rate is shown in Fig. 1.

Results

• Major Issues

o *Lines 213-15: "…enhanced in a dose-dependent manner from 30 to 100 µM, while the heart rates of 10 and 30 µM treatment groups did not show significant difference." The authors claim 30 µM cilostazol increases heart rate, but then claim it and 10µM had no significant difference (from untreated?). The authors should clarify if only 50 and 100 µM caused a significant increase.*

Response

We have followed this reviewer's advice to adopt a new approach to assess the heart rate. Through the ImageJ TSA method we have obtained a new set of results as shown in Figure 1. As compared to the previous set of data generated by hand tally counters, there were slightly different results in terms of multiple comparisons among various concentrations of cilostazol. We believe that the new set of experiments have provided more accurate and unbiased measurements of the heart rate. The revised results have been described in lines 240-55.

o *Lines 213-15: It is unclear what control represents. Authors should compare treated groups to equivalent vehicle control groups.*

Response

In the unrevised version, the control actually refers to the vehicle control. As the reviewer reminded us that DMSO alone may stimulate the heart rate, we have therefore included both blank and vehicle controls in our experiment in Figure 1. Indeed a stimulatory effect of DMSO on the heart rate has been detected at 2 and 3 dpf. Nevertheless, all the cilostazol treatment groups at 3 dpf displayed significant elevation of the heart rate as compared to either blank or vehicle control groups. At 2 dpf, only cilostazol at 100 µM caused a significant effect as compared to the vehicle control. The revised results have been described in lines 240-55.

o *Lines 248 – 250: Authors note that a low DMSO concentration increased measured cortisol*

levels. This accounts for approximately 30% of the claimed cortisol increase caused by 50 and 100 µM, a large amount. Additionally, the authors show that the DMSO control is significantly larger than the 30µM treatment group. The authors must address this difference. It is possible that inappropriate statistics are being used, or there is major batch variation causing false positive results. (See figure 3 comments).

Response

We appreciate this reviewer for critical comments on this experiment. After rechecking, we found a mistake in putting the concentration of DMSO as 0.0019%, which should be 0.18% (0.0018) in all our experiments. We deeply apologize for this error, and corrections have been made by stating the DMSO concentration in the vehicle control in the Materials and Methods, as per this reviewer's another suggestion. We have also amended our results by stating that "the vehicle control containing DMSO at 0.18% caused a 1.4 fold elevation of cortisol production in the 5 dpf embryo as compared to the blank control (Fig. 3)" (lines 289-91).

In addition, we have reworded in the Materials and Methods to address more clearly that "A group of 30 treated embryos pooled as one sample for ELISA was immobilized by ice-cold water and frozen in liquid nitrogen, and 3 ELISA samples were collected from each respective treatment." Hence 90 embryos were used for each chemical concentration, and a total of 540 embryos (generated on the same date; from the same tank of fish with identical date of birth and genetic background) were subject to this ELISA assay. We have also carefully rechecked this set of results to ensure that the statistics are valid. Although it remains unclear why the cortisol amount in the 30 µM cilostazol group was lower than the vehicle control group, it is evident that cilostazol at 50 and 100 µM caused a significant increase of whole embryo cortisol levels (lines 289-296).

We do agree with this reviewer that why cilostazol at 30 µM caused a lower cortisol production than the vehicle control is to be addressed. It is also noted that there was no statistical significance between the blank control and 30 µM cilostazol treated groups. Although quality controls included in the ELISA kit has been followed in our experiment, multiple steps of cortisol extraction prior to the ELISA may also cause variations especially at the range of low cortisol concentrations. Nevertheless, our current data of steroidogenic cell counts and cortisol production still support the effect of cilostazol at high dosages (50 and 100 µM) on steroidogenesis.

o *Lines 281-2: See figure 4 comments. Duplicated data.*

Response

We are indebted to this reviewer for pointing out this mistake. We have rechecked the original data set and verified that it was due to a misplacement of image during figure preparation. As the previous Fig. 4 has been moved to Fig. S2, the panel A of Fig. S2 has been corrected.

o *Lines 283-290: The authors have transformed their neutrophil stain data into a timeseries*

format. They mention a lower number of neutrophils present in all samples by 12 hpa and another upregulation by 24 hpa. The authors use this data to claim cilostazol plays a role in neutrophil retention but not recruitment. However, previous studies using live tracking of neutrophil migration have not seen this resurgence phenomenon (PMID: 16926288) including a real-time live tracking study (PMID: 25763660). The authors should address this discrepancy. The authors should also consider performing a real-time live neutrophil tracking experiment in order to support their claim of cilostazol-mediated neutrophil retention.

Response

Following this reviewer's suggestions, we have performed a live imaging time course study by using the *Tg(mpx:EGFP)* line, and the results were shown in Fig. 4. The previous Fig. 4 results based on the whole mount Mpx activity assay have been moved to Fig. S2 for comparisons. The results of and discrepancy between these two experiments are described in the Results (lines 310-359). We have indeed found that the trend of neutrophil dynamics and the effect of cilostazol treatments appeared divergent between *mpx:EGFP* live imaging and Mpx enzymatic activity staining assays. It is speculated that different genetic backgrounds or various physiological conditions due to experimental processing might cause the discrepancy.

o *Lines 292-304: The authors should make specific mention to the actual data-containing figure 5.*

Response

We thank the reviewer for this reminder. The data-containing Fig. 5A and B have been specifically mentioned in the revised version (lines 372-5).

o *Lines 314-25: Why does DMSO vehicle significantly increase macrophage recruitment over blank control? As with the neutrophil staining, the authors should consider a live real-time analysis of macrophage migration to discuss trends.*

Response

Following this reviewer's advice, we have actually tried to perform a new live imaging experiment by using the *Tg(mpeg1:EGFP)* line. However, the treated fish in our hands started to be visibly unhealthy after 24 hpa, and cannot survive up to 48 hpf. It is speculated that the repeated anesthesia for live imaging on the same batch of embryos may affect the vitality of *Tg(mpeg1:EGFP)* after 24 hpa. Therefore, we apologize for not being able to provide a live imaging study of macrophage behavior. DMSO is known to either enhance or suppress innate immunity in a highly context-dependent manner, depending on the duration and concentration of treatments [PMID: 31987604]. A statement has hence been added to the modified version (lines 395-9).

- Minor Issues
  - *Line 218: "…developing zebrafish may be similar to those on the mammalian adults." Strange phrasing. Rephrase to just say "similar in mammals".*

<u>Response</u>

Lines 257-8 (line 218 in the original version): The sentence has been revised according to this suggestion.

  - *Line 224: "…cilostazol, which enhances cAMP production…". Cilostazol increases intracellular cAMP concentration by blocking PDE3-mediated degradation. It is not known to directly increase cAMP production. Any literature showing cilostazol-mediated cAMP production should be cited.*

<u>Response</u>

Lines 264-5: (line 224 in the original version):

Following this reviewer's suggestion, we have revised the sentence as "...cilostazol, which enhances cAMP production by limiting the cAMP-degrading activity of PDE3..." and a reference has been added.

  - *Lines 240-241: The authors describe the cortisol extraction and quantification procedure in the methods, but they should also briefly mention the experiment again here before discussing results.*

<u>Response</u>

Lines 279-83: (line 239-241 in the original version):

We have revised the sentence as "To further estimate whether the increase of differentiated steroidogenic interrenal cells contributes to higher amount of steroidogenic hormone secretion, whole-body cortisol extraction and cortisol ELISA assays were applied to the larval zebrafish groups treated with different concentrations of cilostazol or control solutions."

  - *Lines 251-53: The authors are trying to compare their cortisol findings with their steroidgenic cell counts in an unclear way. They may wish to create a new last sentence stating something along the line of "increased cortisol levels are consistent with increased number of steroidgenic cells".*

<u>Response</u>

We thank the reviewer for this important suggestion. A sentence has been added as "In summary, the analysis of interrenal steroidogenic cell counts (Fig. 2) and cortisol production (Fig. 3) both support an enhancing effect of cilostazol on steroidogenesis at the concentrations of 50 and 100

µM." (lines 294-6).

Discussion

• Minor Issues

  o *Lines 347-8: "Cortisol is the most important endogenous glucocorticoid in both humans and teleosts". This is a completely subjective statement.*

<u>Response</u>

We have deleted this subjective statement.

  o Line 351: *Missing period.*

<u>Response</u>

Line 428 (Line 351 in the original version): This typo has been corrected.

  o *Lines 398-400: The authors are slightly over stating their findings. Their study does not investigate the persistence of chronic inflammation into zebrafish adulthood, nor does it investigate changes in mental function of the fish. The authors should revise somewhat.*

<u>Response</u>

We have revised the statement as "...the results of our study implicate that exposure to cilostazol during early development may lead to pro-inflammatory adults with changes in mental function, which remains to be investigated in animal models." (Lines 484-6).

Figures

• Major Issues

  o Figure 2:

    ▪ *In panel A, the authors show stained tissue from two zebrafish pigmentless strains with varying cilostazol treatment. The authors have outlined the stained tissue with a red dashed line, but claim to have quantified the number of cells within the tissue in panels B and C. From the images provided, it is unclear how authors were able to resolve individual cells. The bottom center and rightmost images are particularly low resolution and blurry with large overlapping purple areas where individual cells do not appear to be resolvable. The authors must describe the quantification process in the methods. Additionally, the authors should provide sample images of individually identified cells outlined in a similar fashion to panel A, perhaps as a supplemental figure.*

    ▪ *Panels B and C should have a label/title describing from which strain the data is coming from.*

Response

1. Following this reviewer's advice, we have revised Fig. 2 by providing magnified version of the original set of representative images for better resolution.

In the supplemental material Fig. S1, we have also provided examples (based on two sample images in Fig. 2) to show how to resolve individual cells by the multi-point tool of Fuji ImageJ, as well as how to take multiple images from a single sample for resolving individual cells. It is addressed in the legend of Fig. S1 that in the cases where stained interrenal cells on the ventral surface need to be identified by adjusting the focus, multiple images were taken in order to provide sufficient resolution for the whole ventral surface of interrenal tissue clusters (lines 588-90). We have also amended the Materials and Methods to briefly describe how to perform steroidogenic cell counting (lines 165-8).

2. We have added the strain names on panels B and C of Fig. 2.

o Figure 3:

- *In the figure legend, authors claim each sample size is a batch of 30 larvae with 3 samples per treatment group. They also mention either samples or larvae groups are "subject to duplicate measurements". Is each duplicate graphed here as an independent datapoint creating N = 6 samples? This would artificially bias the data. If each histogram is only N = 3, is this enough data points to make statistical conclusions?*

Response

We have addressed in the materials and methods that "...A group of 30 treated embryos pooled as one sample for ELISA was immobilized by ice-cold water and frozen in liquid nitrogen. " And in the legend of Figure 3 that "...Each bar represents the average of 3 independent treated groups, with each group containing 30 larvae and subject to duplicate ELISA tests." Therefore the ELISA result for each treatment is derived from a total of 90 larvae. Altogether, 540 larvae have been used for the ELISA experiment.

o Figure 4:

- ***The authors provide a figure showing that cilostazol treatment causes fewer migrated neutrophils at 24 hpa. However, the 24 hpa provided images in Panel A of 50 and 100 μM treatment have been duplicated. Despite the images being duplicated, the red traced outline is slightly different between the two. The authors should explain and correct the error and ensure that duplicated data was not shared between the 50 and 100 μM dot plots.*
- *452: "50 and 100 μM cilostazol control groups". These are not control groups.*

Response

1. We are grateful to this reviewer for pointing out this mistake. We have rechecked through the original data set and the statistics carefully, and verified that it was due to a misplacement of image for the cilostazol 100 μM/24 hpa group during figure preparation. This error has been

corrected. As the previous Fig. 4 has been moved to Fig. S2, the panel A of Fig. S2 has been revised.

2. “50 and 100 μM cilostazol control groups” has been corrected as “50 and 100 μM cilostazol treated groups” in the legend of Fig. S2 (Fig. 4 in the original version).

o Figure 5 is not mentioned explicitly in the text.

Response

We thank the reviewer for this reminder. The data-containing Fig. 5A and B have been specifically mentioned in the revised version (lines 372-5).
